# Integration intrapreneurship: implementing innovation in a public healthcare organization

**DOI:** 10.1186/s13731-022-00248-x

**Published:** 2022-10-04

**Authors:** Perrin Moss, Nicole Hartley, Trevor Russell

**Affiliations:** 1 Integrated Care, Children’s Health Queensland Hospital and Health Service, Brisbane, Australia; 2 School of Business, The University of Queensland, Brisbane, Australia; 3 School of Health and Rehabilitation Sciences, The University of Queensland, Brisbane, Australia; 4 RECOVER Injury Research Centre, The University of Queensland, Brisbane, Australia

**Keywords:** Intrapreneurship, Healthcare, Telementoring, Integration, Project ECHO, Australia

## Abstract

**Aim:**

As global events impact the way organizations operate and innovate in response to regional, workforce and consumer needs, the concept of intrapreneurism is attracting growing interest from policymakers and executives, particularly within the healthcare sector. The aim of this study was to capture the key learnings from the implementation of a telementoring pilot, to understand how intrapreneurship can embed innovation within an established organization to effect more integrated healthcare.

**Purpose:**

A qualitative approach was used with a phenomenological lens to explore the key learnings of the Project ECHO^®^ (Extension for Community Healthcare Outcomes) pilot implementation to provide an understanding of what the project team’s strategies and tactics were during the process of embedding a new business innovation. The implementation and piloting of Project ECHO^®^, a telementoring model, in a large-scale public healthcare organization in Queensland, Australia, was investigated as an exemplar of integration intrapreneurship.

**Findings:**

Through an inductive approach, this qualitative study found the implementation of the Project ECHO^®^ pilot had specific dimensions and strategies/tactics which were exemplars of intrapreneurism. The organizational context and workforce characteristics described in this study presented new knowledge of how intrapreneurs implemented an innovation to address fragmentation of healthcare service delivery, professional isolation and instances of low-value care. This research contributes to a better understanding of the strategic and tactical approaches to implementing intrapreneurial innovations within a public healthcare organization, with learnings that can be adapted by intrapreneurs in other contexts.

## Introduction

Organizations within the healthcare sector are susceptible to economic pressures and as a result are prone to continuous and transformational change (Melder et al., 2018; Nicholson et al., 2014). This landscape has contributed to a climate ripe for innovative change for healthcare organizations at a local, regional, and global level. Opportunistic and motivated individuals and organizations continue to develop new products and services to address the evolving needs of the workforce and community. Healthcare executives and decision-makers can facilitate effective service redesign and innovation by empowering and nurturing emerging leaders and clinicians (Melder et al., 2018). Executives and other decision-making leaders who encourage innovation and disruption actively create an organizational culture and pipeline process of identifying and growing new value (Antoncic & Hisrich, 2003). Employees within these organizations that can be characterized by their motivation to proactively identify and seize opportunities to acquire resources (human and capital) to implement innovations, change, or departures from the status quo are considered intrapreneurs (Altinay, 2004; Falola et al., 2018; Heinze & Weber, 2015; Park et al., 2014). The contextual landscape of public healthcare organizations is conducive to fostering cultures where the concept of intrapreneurship can be mobilized by the workforce to innovate and improve the delivery of healthcare services.

Innovative approaches to delivering education and case-based learning has become essential for large-scale organizations striving to achieve strategic and operational objectives, especially in the healthcare sector. This has been highlighted in more recent times in light of the global COVID-19 pandemic, where healthcare organizations have been forced to rapidly adopt virtual or telementoring platforms to sustain and support the learning needs of their workforce. Employees within these organizations have moved from first responding to the management and containment of the virus, to adopting new ways of delivering services beyond the outbreak. The landscape within this sector is now, more than ever one where organizations are juggling the need to innovate while remaining focussed on delivering high quality patient care and maintaining financial sustainability (Oderanti & Li, 2018).

Global events, including the more recent COVID-19 pandemic, have driven the cumulative growth in organizational utilization of telementoring training programmes as a mode of specialized workforce development and education delivery due to the distinct advantages of ameliorating geographic barriers and now more so to ensure compliance with social distancing etiquette (Gleason et al., 2020; Kay & Spicer, 2020). Telementoring is a virtual learning approach where real-time videoconferencing technology is used to provide a teaching and learning environment between content knowledge experts (based at central hubs) and participating frontline providers (working at multiple spoke sites) which contribute their local context expertise (Lewiecki et al., 2017). Telementoring has become common within the public sector where government agencies have seen community expectations evolve, resulting in demand for higher quality, more variety and lower cost services (Mohsen et al., 2019; Wanna et al., 2015). A number of telementoring models have been developed in efforts to generate value, productivity and efficiency in the public healthcare sector (Christian & Andreas, 2019; Clark & Goodwin, 2010; Oderanti & Li, 2018; Socolovsky et al., 2013; Tuerk, 2015). Project ECHO^®^ (which stands for Extension for Community Healthcare Outcomes) is an established model of telementoring with a track record for improving health outcomes in North America (Arora et al., 2011). However, there is a gap in the literature regarding how innovative models such as Project ECHO^®^ are implemented within established organizations.

The aim of this study is to capture the key learnings from the implementation of a telementoring pilot, to understand how intrapreneurship can embed innovation within an established organization to effect more integrated healthcare. Intrapreneurism is an organizational phenomenon which can be defined as entrepreneurship, or the process of discovering and advancing opportunities to create value through innovation, that occurs within existing organizations (Antoncic & Hisrich, 2003). Intrapreneurs are therefore most often employees within these organizations rather than entrepreneurs who are generally associated with new start-up ventures. This study will explore intrapreneurial strategies and tactics used by the project implementation team throughout the Project ECHO^®^ pilot to embed the intrapreneurial dimensions of the innovation within Children’s Health Queensland Hospital and Health Service (CHQHHS), a large-scale paediatric public healthcare organization in Queensland, Australia.

Intrapreneurism is attracting growing interest from policymakers and executives within the healthcare sector at a regional and international level (Brown et al., 2014; Carpenter et al., 2018; Mundy & Hewson, 2019). This has been largely a result of evolving service needs of ageing populations and more recently, an increasing prevalence of chronic diseases that place pressure on healthcare organizations to sustain the delivery of high quality and economically viable healthcare services (Mundy & Hewson, 2019; Oderanti & Li, 2018). These factors have created a landscape that is more conducive for intrapreneurs to explore and implement innovations that are aimed at integrating healthcare.

Prior research in this field (Moss et al., 2020) has explored the conscious decision-making processes of healthcare executives and the factors that influence these gatekeepers to invest in integrated healthcare innovations. Key findings from this and similar studies (Enslin, 2010; Falola et al., 2018; Gawke et al., 2019; Guven, 2020; Heinze & Weber, 2015; Neessen et al., 2019; Park et al., 2014), indicate that intrapreneurial champions within organizations can positively influence decision-making processes resulting in organizational commitment to, and financial support of innovative pilots. Aside from these studies, empirical investigation of intrapreneurship within the Australian public sector is limited (Gapp & Fisher, 2007; O'Connor et al., 2007), with few studies exploring how intrapreneurial pilots are successfully implemented. With this in mind, this study sought to capture the key learnings from the implementation of a telementoring pilot to understand how intrapreneurship can embed innovation within an established public healthcare organization to effect more integrated healthcare. The findings of this study provide an illustration of intrapreneurship within the Australian public healthcare sector that can be replicated by other organizations across sectors at a regional level. Further, this study provides new knowledge in the field of integration intrapreneurship by exploring conceptual elements of intrapreneurial theory that have been identified in an Australian integrated care pilot.

### Conceptual background for intrapreneurism

The concept of intrapreneurship has evolved in the management literature over the last 40 years as a distinct theoretical construct to better understand the behavioural intentions of employees seeking to innovate within established organizations. To this point, Antoncic and Hisrich’s (2003) seminal study clarified the intrapreneurship concept and identified eight dimensions that characterize intrapreneurial processes, innovative activities and orientations to frame how intrapreneurs approach their particular innovation (see Table 1). Intrapreneurship can be illustrated as emergent behavioural intentions and behaviours that relate to departures from the status quo within existing organizations. While these dimensions highlight characteristics, the concept of intrapreneurism refers not only to the creation of new business ventures, but also to other activities and orientations that involve innovation (Antoncic & Hisrich, 2003). Within the context of the healthcare sector, the dimensions of intrapreneurship provide a useful lens by which to understand how innovation and integration within organizations and systems can be achieved. This study will use the dimensions to highlight and showcase exemplars gleaned from a telementoring pilot implementation through an inductive approach.

**Table 1:** Eight dimensions of intrapreneurship, adapted from Antoncic and Hisrich (2003)

Intrapreneurship dimension and definition
1. New ventures: Creation of new autonomous or semi-autonomous units or firms
2. New businesses: Pursuit of and entering into new business related to current products or markets
3. Product/service innovativeness: Creation of new products and services
4. Process innovativeness: Innovations in production procedures and techniques
5. Self-renewal: Strategy reformulation, reorganization, and organizational change
6. Risk taking: Possibility of loss related to quickness in taking bold actions and committing resources in the pursuit of new opportunities
7. Proactiveness: Top management orientation for pioneering and initiative taking
8. Competitive aggressiveness: Aggressive posturing towards competitors/sector/system manager(s)

Copyright Emerald Group Publishing Limited

While intrapreneurial ventures can be defined by the eight dimensions in Table 1, the pursuit of the innovation itself can often fail without strategic insight and tactical considerations (Oderanti & Li, 2018). For intrapreneurism to succeed, particularly to achieve more integrated healthcare at a systems and organizational level, intrapreneurs must adopt tactical approaches to implementation (Heinze & Weber, 2015).

To complement these dimensions, Heinze and Weber (2015) identified eight strategies or tactical approaches which intrapreneurs can use to enable their innovations to be adopted at the organizational level. The strategies and tactics recommended by Heinze and Weber (2015) were shown to create and strengthen organizational free spaces aligned with the relevant dimensions of intrapreneurial innovations. Intrapreneurs can select and tailor these individual strategies/tactics to leverage the capacity required to develop and integrate the innovative change within the broader organization (Heinze & Weber, 2015).

Antoncic and Hisrich’s (2001, 2003) work over the last two decades has been widely cited as providing a reliable criterion to identify an innovation as being intrapreneurial (Antoncic & Antoncic, 2011; Fitzsimmons et al., 2005). To expand on this conceptual framework, the strategies/tactics purported by Heinze and Weber (2015) provide a targeted and customizable approach that individuals or teams have referenced in almost 30 other studies to achieve successful intrapreneurial ventures within an organizational context. These approaches are defined in Table 2. Published literature has found that where intrapreneurial teams pursued one or more of the dimensions as cited by Antoncic and Hisrich (2003) in partnership with strategic/tactical posturing as presented by Heinze and Weber (2015), their implementations were well-received within the implementing organization (Amini et al., 2018; Burton-Jones et al., 2020; Di Lu & Heinze, 2021; Dimitratos et al., 2012; Eriksson & Ujvari, 2015; Fitzsimmons et al., 2005; Gapp & Fisher, 2007; Garbutt et al., 2019; Gerards et al., 2020; Grayson et al., 2014; Hoogstraaten et al., 2020; Melder et al., 2018; Neessen et al., 2019). Given the aim of this study was to understand how intrapreneurship can be harnessed to embed innovation within an established organization to effect more integrated healthcare, we sought to assess key learnings from the intrapreneurial team against intrapreneurial approaches espoused in the literature. Consequently, the results from this study highlight how integral it is for intrapreneurs to consider the relevant dimensions of intrapreneurship when designing targeted strategies in their pursuit and implementation of innovation. We also highlight practical aspects of intrapreneurism that can be replicated by organizational decision-makers and leaders to innovate and change the status quo. There is a need to understand how intrapreneurial ventures like implementing Project ECHO^®^ within dynamic sectors such as healthcare can support organizational advancement to meet market needs.

**Table 2:** Strategies recommended to support intrapreneurs, adapted from Heinze and Weber (2015)

Strategy and definition
1. Leverage status in the institutional field: Where intrapreneurs build credibility in their broader profession by taking on roles that carry status and influence, including academia, associations, boards. This enhances their credibility and visibility internally to influence and legitimize the need for change
2. Gain proprietary jurisdiction over resources: Where intrapreneurs directly and indirectly acquire and administer external funding sources to build and extend their programme of work, thereby reducing dependence on and competition for resources and decision structures within the organization
3. Create trading zones: Where intrapreneurs identify, prioritize, and participate in/attend spaces/forums where their ideas and knowledge can be exchanged in a low-stakes environment between internal stakeholders. This is particularly focused on engagement opportunities that are associated with learning and respectful inquiry rather than decision-making and endorsement processes. The intrapreneur may repurpose the agenda or direction of these forums to achieve mutual learning that aids the change objective(s)
4. Build a pipeline: Where intrapreneurs systematically build a core following of individuals that engage in the change dialogue and contribute to mobilizing the change
5. Use experimentation to build capacity: Where intrapreneurs can learn, reflect on, and refine their approaches, to improve effectiveness, to enable increased responsiveness and targetability for future opportunities. Experimentation supports the intrapreneur to learn how to improve their execution and gain greater acceptance of their change. Experiments can incorporate the refining of concepts, templates or resource development for future utility, and other similar building block activities that enhance the intrapreneur’s sensitivity to time-limited opportunities
6. Establish formal free spaces with endowed resources and status (Requires strategies 1 and 2. When done well, this tactic also enhances the effectiveness of strategies 3, 4, 5): Where intrapreneurs can establish and formalize business units within the organization. This can include establishing dedicated roles, office accommodation and access to necessary facilities and ancillary supports
7. Create and exploit opportunities for ongoing change: Where intrapreneurs maintain constant focus on horizon scanning activities to identify additional opportunities to bolster change efforts. This can include acquiring additional funding, fostering new partnerships, technological or operational enhancements, and personnel/organizational/system/political events
8. Extensive and diverse representation/membership at multiple organizational forums/communities: Where the intrapreneur integrates their role and presence across several of the organization’s forums/communities. This is to sustain regular engagement with stakeholders to bed down the change and identify improvement/expansion opportunities

Copyright INFORMS, Institute for Operations Research and Management of Science

### Project ECHO^®^ in Queensland—new evidence of intrapreneurism in a public healthcare organization

The 2016 pilot implementation of a telementoring model called Project ECHO^®^ (Arora et al., 2010; ECHO Institute, 2020a), in Queensland is presented in this study as new evidence that intrapreneurship is being actively pursued in public healthcare sector organizations to innovate and improve the way organizations provide more integrated healthcare. The Project ECHO^®^ pilot was led by a general practice liaison officer and project manager employed at CHQHHS, the state’s tertiary paediatric provider (Children’s Health Queensland Hospital & Health Service, 2019). The aim of the pilot was to achieve more integrated healthcare for children and young people across the healthcare continuum using the model to connect frontline providers across urban, regional, rural, and remote centres with the metropolitan-based tertiary healthcare centre. It was hypothesized that providing a telementoring service, using the ECHO model™, to mentor and upskill frontline health and other professionals across Queensland would achieve more integrated and comprehensive healthcare to patients in their local communities (Carter et al., 2019). By facilitating virtual access to interprofessional advice and support from mentors and colleagues in complimentary settings such as the tertiary paediatric hospital, there was thought to be potential to increase capacity for local service provision while reducing demands on the single paediatric facility as the default provider of all services (Carter et al., 2019). The project implementation team thought that this pilot would lend itself to being quickly scaled across other prevalent health conditions that were well-suited to a telementoring approach.

The Queensland-first pilot created a virtual ECHO^®^ Network to support and enhance regional capability of service providers delivering healthcare to children with stable Attention Deficit Hyperactivity Disorder (ADHD). The ADHD pilot was used as a catalyst for the organization to consider using Project ECHO^®^ more broadly to innovate and address service demands. Globally, the purpose of healthcare organizations using the ECHO model™ has been to improve access to healthcare for underserved communities, or priority populations who experience barriers to accessing best practice care locally (Arora et al., 2010; Furlan et al., 2019; McBain et al., 2019; Zurawski et al., 2016). Organizations which have implemented the ECHO model™ operate as ECHO^®^ ‘hubs’ to leverage and scale their scarce internal workforce ‘content’ expertise by providing freely accessible, virtual case-based education in ‘communities of practice’ (Carpenter et al., 2018) to support peer to peer learning amongst frontline context experts in community ‘spoke’ centres located anywhere (Arora et al., 2010). The key point of difference between the ECHO model™ and other models of telementoring is the case-based learning component where spoke participants present on their own case scenarios to receive advice and support from colleagues participating in the ECHO^®^ Network (Anderson et al., 2017; Damian et al., 2020; Furlan et al., 2019; Socolovsky et al., 2013; Tantillo et al., 2019; Tosi et al., 2020; Zurawski et al., 2016). Evidence from North America has shown ECHO^®^ Networks enabling the local management of complex cases by frontline providers supported by specialist teams to reduce barriers to accessing best practice healthcare and experiencing improved health outcomes at a regional level (Arora et al., 2011). In the CHQHHS scenario, the project implementation team consisted of four employees, who engaged clinicians from within the organization to leverage their paediatric content expertise to facilitate the pilot innovation as ECHO^®^ Network panellists. These panel roles were fulfilled by a team of medical and allied health clinicians, educators, and parent representatives, with spoke participation from context experts including general practitioners, psychologists, and guidance officers throughout Queensland. This Queensland-based team launched the first paediatric ECHO^®^ Network in Australasia in May 2017. From the ADHD pilot, early adopters from within the organization partnered with the project team to rapidly expand the model’s use within the organization across a wider range of paediatric health conditions. These included behaviour and mental health, diabetes, foot anomalies, obesity, palliative care, persistent pain, and refugee health.

In intrapreneurial terms, the implementation of the Project ECHO^®^ hub at CHQHHS to deliver the ADHD ECHO^®^ Network represented a clear departure from the customary provision of direct patient care in a hospital outpatient setting. The aim of providing the ADHD ECHO^®^ Network was to provide participants in every region across Queensland with access to the telementoring platform to gain new knowledge and confidence in managing children with stable ADHD in their local communities through virtual case-based learning sessions using the ECHO model™. This change in practice was seen by the organization as a way to integrate the access and delivery of a broad array of healthcare services by decreasing frontline providers defaulting to referral of children to hospital outpatient services for sub-specialist management which was common practice nationally (Mitchell et al., 2015; Shaw et al., 2002; Thomas et al., 2013). This intrapreneurial approach by the project implementation team manoeuvred leaders within the organization to consider the pilot as a catalyst for ongoing change and innovation in other areas of the business. This in turn served as a tactic for the project team to embed the ECHO model™ within the organization as a business-as-usual solution to address service demands.

## Materials and methods

### Method design

This study used a qualitative approach with a phenomenological lens (Bevan, 2014; Glendinning, 2008) through primary in-depth interviews with the core project implementation stakeholders, and a secondary desktop data analysis. Consistent with intrapreneurism being an organizational phenomenon, a descriptive phenomenological approach to interviewing was used to discover and contextualize the experience of the intrapreneurs involved in the pilot (Antoncic & Hisrich, 2003; Bevan, 2014). The interviews and secondary data collection process was aligned to descriptive phenomenological human scientific research (Englander, 2012). This phenomenological approach was the total method for research, not just an area of focus during the data analysis. The structured style used during the interviews applied questions that were based on contextualizing participant’s experiences, captured in relation to the Project ECHO^®^ pilot in Queensland as a phenomenon, and sought clarification of these lived experiences through their perspectives (Bevan, 2014).

### Interview participants

This research was conducted by the author (PM) with three CHQHHS staff who made up the core project team. This group were categorized as the intrapreneurs within the organization who had key involvement in the first pilot implementation of Project ECHO^®^ in Queensland. Participant sampling was purposive and exhaustive, with inclusion criteria relating to the participant’s core involvement during the implementation, therefore no other participants were pertinent. The sample was also the entire stakeholder group of project roles who had knowledge of and involvement in the implementation process, aside from the author (PM). The author (PM) was a fourth core member of the project team and had cumulative knowledge of the implementation. For this reason, the total sample of three interview participants achieved data saturation as there were no other informants involved in this implementation. Each of the participants had worked outside of a metropolitan centre and in the primary healthcare sector. These career demographics enhanced the participants’ understanding of how the telementoring model could be best used by the organization to support capacity building amongst the primary healthcare workforce across Queensland to integrate care (Moss et al., 2020).

### Secondary desktop data

The secondary data sources included supporting project management documentation and observational field notes taken during the primary interviews (Tong et al., 2007). The secondary data sources (Integrated Care Innovation Fund (ICIF) grant proposal, project plan, Gantt chart, implementation diary, and pilot evaluation report) were reviewed in parallel to observational field notes to reconcile and validate statements made by the interview participants. This data was able to be mapped to various dimensions and strategies/tactics of intrapreneurism to highlight where alignment to the literature could be demonstrated.

### Data collection

This research was conducted on CHQHHS premises at the Centre for Children’s Health Research in South Brisbane, Queensland. Interviews ran for approximately one hour each and were voice recorded, transcribed, and sent to participants for member checking. The semi-structured interview questions elicited participants' reflections of the lessons learned during the pilot implementation, including tactical successes, failures, and future opportunities. The author (PM) made detailed written field notes during the interviews which were also analysed. The field notes extended to related observations that took place during and immediately outside the interview encounters, e.g., incidental conversations with participants.

Data were collected in an identified manner, but in these research findings, the data have been reported in de-identified form with participant codes. Secondary data resources (ICIF grant proposal, project plan, Gantt chart, and implementation diary) which came to light during interviews were subsequently provided by the participants to the author (PM) at their discretion.

### Data analysis

The interview transcripts were analysed using NVivo 12, a qualitative analysis software program (QSR International, 2020) to develop a thematic framework. A thematic analysis was conducted to explore whether the pilot implementation had any dimensions of intrapreneurism present. An inductive approach was taken following semi-structured interviews with research participants to develop an explanation for the patterns and relationships that were found in the interview transcripts. This inductive approach was employed to draw out unique findings in the analysis to showcase the Project ECHO^®^ pilot implementation as an exemplar of intrapreneurism within the healthcare sector. These findings were such that could be replicated in other organizations—particularly in the health and human services sectors where employees’ desire to innovate can be held back by organizational decision-makers’ focus on meeting service demands and financial sustainability pressures. Other documentation (ICIF grant proposal, project plan, Gantt chart, implementation diary) provided by interview participants were also reviewed in a desktop analysis. A thematic framework and coding guide were developed during the analysis process. The documentary evidence was analysed independently following analysis of the interview transcripts to substantiate and triangulate the interview findings.

Three co-investigators also conducted a content analysis on de-identified transcripts to triangulate the data, to contextualize and frame the findings. This was a blind review between each of the co-investigators to support consistency in identifying major and minor themes given the small sample size. The data analysis sought to identify which dimensions and strategies/tactics of intrapreneurship were most relevant in the large-scale hospital setting in which Project ECHO^®^ was implemented.

The author (PM) also employed a process of bracketing (Bevan, 2014), whereby first-hand knowledge of the Project ECHO^®^ implementation phenomenon was set aside to ensure the interview dialogue remained faithful to the descriptions of experience of the participants being interviewed, and was accepted that this was how they described their experiences and perceptions. This assisted in maintaining a fundamental level of validity in abstaining from the use of personal knowledge to steer questioning of any of the interview participants (Bevan, 2014). Examples of learnings will be discussed in the results section.

The CHQHHS and University of Queensland Human Research Ethics Committees approved this study under reference number: LNR/18/QCHQ/44762.

## Results

The aim of this study was to explore the strategic/tactical approaches of an intrapreneurial team to capture the key learnings from the implementation of the Project ECHO^®^ pilot. This was to better understand how intrapreneurship can embed innovation within an established organization to effect more integrated healthcare. The interview theming and documentary analysis findings are presented below to illustrate how dimensions of intrapreneurism were embedded using strategies/tactics by the project implementation team. Key learnings that can be adopted and directly applied in other settings are explored under each strategy/tactic throughout this section.

While aspects of the 2016-onward pilot implementation aligned well to many of the dimensions of intrapreneurship, Project ECHO^®^ was in this scenario considered an exemplar of a new venture and new business (Dimensions 1 and 2) (Antoncic & Hisrich, 2003). The interview data which will be discussed below will provide context around the key learnings and highlight examples of the strategies/tactics used by the intrapreneurial project implementation team to discuss how they were integral in the success of Project ECHO^®^ pilot implementation. This research will illustrate that intrapreneurism was not a single experience and continued as a series of behaviours and actions of embedding and maturing the innovation throughout the implementation journey. The chronological series of events captured in the data indicated the dimensions of intrapreneurship were more pronounced or formally established during the early planning phase. However, the strategies/tactics employed by the intrapreneurs to embed the innovation were continuous throughout the implementation phase.

### Dimensions of intrapreneurship—organizational-level learnings from implementing Project ECHO^®^

Between January and June 2016, the general practice liaison officer and project manager collaborated to develop a clearly articulated project plan and secure grant funding to establish a Project ECHO^®^ hub within CHQHHS. This new venture (Dimension 1) (Antoncic & Hisrich, 2003) involved adopting a licensed telementoring model from the United States and essentially creating a franchise hub within CHQHHS (Altinay, 2004; ECHO Institute, 2020b). The proposal to pilot a telementoring model to build capacity in the primary healthcare sector workforce constituted a significant change in direction from the direct service provider business-as-usual activity of the hospital and health service organization. At the time of implementation, there was no suitable business model for sustainably integrating the ECHO model™ within the Queensland healthcare sector (Oderanti & Li, 2018). As a result, the new venture relied on the project team’s ability to attract ongoing competitive and philanthropic grant funding. The organization required the project team to operate as an autonomous business unit while piloting the innovation to delineate from core, funded, business-as-usual activity (Moss et al., 2020). The organization’s executive decision-makers saw the new venture as a way to integrate a range of existing service objectives within the fragmented public healthcare system (Moss et al., 2020).

The project implementation team’s ambition to become the first Project ECHO^®^ hub in Queensland was underpinned by CHQHHS’s integrated care agenda to use the model to establish a new business within the public healthcare organization to provide paediatric-focussed telementoring across Queensland (Dimension 2) (Antoncic & Hisrich, 2003). The objective of establishing a pilot ADHD ECHO^®^ Network was to test the viability of the model to deliver a virtual community of practice aiming to empower General Practitioners and other primary care providers to manage children with stable ADHD in their local communities. This aim was thought to decrease demands on specialist paediatric outpatient departments in Queensland hospitals and achieve a more integrated way of delivering healthcare across the service continuum and reduce geographic barriers to accessing care. Implementing and piloting the ECHO model™ was embarking on a new approach to achieve healthcare integration between primary, secondary, and tertiary healthcare service providers, as well as educators and professionals from other human service sectors. This new business operated by providing telementoring, or virtual education and learning environments (as a new product/service) to facilitate interprofessional ways of providing frontline service providers with access to education and learning through case-based mentorship. This way of working across the healthcare continuum in new markets had not previously been explored by CHQHHS.

### Strategies and tactical approaches to intrapreneurship—organizational learnings from implementing Project ECHO^®^

The dimensions in Table 1, particularly dimensions 1 and 2, highlight how the implementation of the ECHO model™ as an innovation was uniquely intrapreneurial and a departure from the organization’s current state. However, it is essential to understand and explore the complex strategies/tactics that were used by the intrapreneurial project implementation team throughout the pilot to successfully embed the innovation within a large-scale public healthcare organization.

A series of exemplars that were identified during the project implementation phase are presented in this section to showcase where the intrapreneurs employed strategies/tactics to embed the Project ECHO^®^ innovation (Heinze & Weber, 2015). These strategies/tactics employed by the project implementation team were agile and responsive to the emergent organizational landscape. Direct quotations have also been included to provide context around opportunities, challenges and overall organizational learnings elicited from the interview participants during the implementation which were interrogated against current understandings in the literature (Heinze & Weber, 2015). This section will present the Project ECHO^®^ implementation in Queensland as an exemplar to better understand how intrapreneurship can embed innovation within established organizations to effect more integrated healthcare.

#### Leverage status in the institutional field (Strategy 1)

During the implementation phase there were several examples where the project team leveraged their status within the institutional field. Firstly, in late 2016, the general practice liaison officer was promoted to become the medical director for integrated care within the organization. This was a unique and new leadership role established by CHQHHS to build on the track record of the individual and promote the credibility of integrated approaches to healthcare innovation in Queensland. This leveraging of status in the institutional field was amplified by CHQHHS in 2017 becoming a founding member of the International Foundation of Integrated Care (IFIC) in Australia. Further to the IFIC membership, a regional integrated care conference was held in Brisbane in early 2017 where the project team negotiated for Professor Sanjeev Arora, the Project ECHO^®^ founder, to present a keynote plenary address. Professor Arora’s international status was leveraged to showcase his lived experience realizing the ECHO model™’s potential in improving healthcare outcomes and system integration (Arora et al., 2011), as well as his confidence in project team’s potential to integrate care regionally across the South Pacific (Newcomb & Moss, 2018). The interview participants reported that it was ‘*key to prioritize the relationship that you have with other people in the ECHO community*’ (Participant 1) in order to promote CHQHHS’s first-mover advantage in adopting the model in Queensland.

In absence of empirical evidence of the model’s success in Queensland, the organization leveraged the pre-existing international reputation of the ECHO model™ in parallel to the organization’s strategic role as a state-wide service provider for paediatric healthcare to raise profile for establishing the first hub in Queensland (Arora et al., 2011; Children’s Health Queensland Hospital & Health Service, 2019). *‘It’s been a battle to get our internal specialists to embrace it [the ECHO model]. And so that has meant more work for us because we’re constantly trying to convince people that this is a great thing to be part of and a really efficient way to deliver their services, but we have to drive that and so, if there was some way that we could just get that message across, and they organize the other individuals within the organization [to] drive it amongst their own teams, I think that would be more sustainable’* (Participant 3).

The ECHO model™ highlighted to executives within CHQHHS and the partnering Primary Health Networks (PHNs) that this model could contribute to reducing instances of low-value care—where scarce, expensive tertiary paediatric resources within the organization were providing limited or poorly utilized healthcare interventions to small numbers of the overall state population. The ECHO^®^ ethos of ‘all teach, all learn’ served as a key point of difference from other existing workforce development and education models as it explicitly focused on partnerships and a non-hierarchical approach to telementoring amongst all participants. The project implementation team’s fidelity to the ECHO model™’s learner-centric approach to identifying the learning objectives of the participant audience was demonstrated by curating relevant content experts on the ECHO^®^ Network panels to address the participants’ learning objectives in a collaborative and interactive forum (De Witt Jansen et al., 2018; Hauer & Quill, 2011; Lewiecki et al., 2017; Serhal et al., 2018).

#### Gain proprietary jurisdiction over resources (Strategy 2)

Through the acquisition of ongoing grant funding and own-source revenue, the project implementation team were able to gain and retain proprietary jurisdiction over resources. *‘I think the lesson here was the importance of communication, the importance of getting everything in writing, not taking anything for granted’* (Participant 3). This enabled the team to retain autonomy over the strategic objectives of the pilot to integrate care, recruit project and hub operational staff, fit-out of dedicated teleECHO™ clinic rooms and purchase ICT equipment. *‘We need[ed] to advocate, to get that funding’* (Participant 2). The grant funding provided a secure foothold for dedicated resourcing to test the model with fidelity in an incubation period within the organization. Early adopters from within the organization also contributed in-kind labour resourcing to pilot specific additional ECHO^®^ networks focussed on other paediatric health conditions in the short-term to investigate the feasibility to effect integrated care at a state-wide level.

#### Create trading zones (Strategy 3)

In addition to jurisdiction over resources, the team also created trading zones over the course of the implementation. These trading zones were spaces where the project implementation team used existing forums and venues within the organization to facilitate the exchange of ideas and knowledge between colleagues in low-stakes interactions. *‘Understanding of the scope of ECHO, how big picture it is, and what it can achieve [required] some dedicated time as a team to actually really think about and appreciate why we follow the Anatomy of an ECHO [resource], and how actually learning loops build expertise over time, and why we really need to respect the experiences and expertise of the participants in the room, and, do a learning needs assessment’* (Participant 1). This was done by participating in existing and establishing new forums including grand rounds, departmental and executive committees to raise awareness and share their innovative ideas, opportunities to collaborate and interim outcomes with internal stakeholders in non-threatening context. *‘We know from our colleagues around the world that the model can be really effective in things like the child safety system and in offender health and upskilling people in prison settings to provide a level of healthcare to their fellow inmates, and in agricultural contexts as well’* (Participant 1). The project team also leveraged the organization’s partnerships with other agencies at internal and external forums to pique the interest of stakeholders to disseminate a greater understanding of the innovation, answer questions and engage in collaborative dialogue. This diversified, cross-sector and regional approach to networking opportunities opened dialogue with prospective collaborators, investors, and researchers. ‘*It improves the opinions that you're getting in and it makes for a much more sustainable long-term solution*’ (Participant 2).

#### Build a pipeline (Strategy 4)

These internal trading zones facilitated a soft entry for like-minded or early adopters from within the organization which led to the rapid expansion of new ECHO^®^ Networks within the organization (Newcomb & Moss, 2019). By doing this, the team facilitated opportunities for using experimentation to build capacity and interest. In harvesting the buy-in and enthusiasm generated in the trading zones, the project team were able to build a pipeline of willing stakeholders, often departmental leaders and clinical champions, who were prepared to commit to and invest in piloting ECHO^®^ Networks focused on their area of content expertise. *‘There’s been a real willingness to embrace new things and to try things out to see how they work. And that has been, in my opinion, a reason that we have had a lot of great successes realized with the funding that we’ve [acquired]. In the ECHO Networks that we’ve been able to start, grow and expand, we've seen movement into the Department of Education [audience] and growth in that area’* (Participant 1). While this enthusiasm grew, it also facilitated the project team to train a core following of internal staff that were sufficiently engaged in the methodology, rationale, and advocacy of the innovation’s new logic throughout the organization. *‘In the [ECHO Networks] that combined education staff and health staff, that relationship has really improved and the, the interprofessional respect has really grown. So that’s fantastic. I can see that even our specialists are surprised at the skills of [participants]’* (Participant 3). To consolidate this pipeline, the project team iteratively refined and improved the functions of the established hub management team and network panel roles that were recruited to through open merit and targeted recruitment processes.

The interview participants acknowledged that this evolving pipeline came to fruition following the ECHO^®^ Superhub designation in 2018 (Moss et al., 2021). This was when the project team was authorized to commence delivering ECHO^®^ Immersion training from 2019 to license and accredit other teams (internal and external organizational teams) to replicate the ECHO model™ independently. The interview participants reported that CHQHHS’s Superhub designation was a result of the ECHO Institute™ at the University of New Mexico (the licensor) considering the pilot implementation and fidelity assurance to have been of a calibre that was of strategic importance to the model being scaled across the Asia–Pacific Region. ‘*We were very flexible and very good at working around [implementation] problems to come up with solutions, which is no doubt why we’re still here!*’ (Participant 3). This created a new parallel pipeline to increase adoption of the ECHO model™ regionally throughout the South Pacific beside existing CHQHHS ECHO^®^ Networks, which postured the organization’s pursuit of excellence in innovating paediatric healthcare at scale.

#### Use experimentation to build capacity (Strategy 5)

The cumulative experience in using the ECHO model™ since 2016 provided the project team with ongoing opportunities to use experimentation to build capacity and interest, while refining and embedding the innovation within the organization. In doing this, the project team localized existing, and developed new, tools and resources to support the model’s implementation success and adoption as a new logic to integrate care within CHQHHS, and subsequently more broadly across the region.

Low rates of General Practitioner participation during the ADHD pilot led to quickly adopting more interprofessional recruitment strategies for participant audiences in all future networks. This shift in tactic broadened the diversity of professional groups represented as learners in the virtual communities of practice. This experimentation was cited as providing several key benefits including sustaining participation numbers, broadening expertise being contributed by context experts across multiple geographic locations, and more constructive solutions being recommended during the case-based learning portion of each telementoring session. *‘I really perceive an [ECHO] Network like Kids and Teens Behaviour and Mental Health as having huge capacity to drive cultural change over time and practices around management of certain conditions and understandings of when children need to be referred for further care’*—Participant 1.

The organization used the piloting of the Project ECHO^®^ model to experiment with telementoring to integrate healthcare and build workforce capability through interprofessional working. *‘We’re constantly trying to convince people that this is a great thing to be part of and a really efficient way to deliver their services’* (Participant 3). The ECHO model™ was cited by interview participants as providing opportunity for continuous innovation and learning within and beyond the organization. Other key examples of experimentation included iterative curricula development, refinement of the learning needs assessment approach, and key fidelity and communication toolkit resources being adapted to the Australian context. *‘Where [else] do you have all of those professions represented? [Those] people can hear from one another and learn about what care for these kids looks like in different contexts. [It] can be really effective’* (Participant 1).

#### Establish formal free spaces with endowed resources and status (Strategy 6)

To compliment the proprietary jurisdiction over resources, creation of trading zones and experimentation, the project team were also successful in establishing formal free spaces with endowed resources and status. This was achieved whereby the project team created and recruited ECHO^®^-specific roles and portfolios of work within the organizational structure under the leadership of the Medical Director of Integrated Care. This also included the dedication and fit-out of two teleECHO™ clinic rooms and ICT equipment to run the ECHO^®^ hub operations. The formal roles, portfolios of work, accommodation and facilities infrastructure provided validation and governance oversight within the organization that there was ongoing executive sponsorship of the innovation being adopted as business as usual. *‘The PHNs were [also] very excited about ECHO and very keen to participate, and to support us [project implementation team]’* (Participant 3) to leverage the model to engage with and contribute to workforce development priorities across the primary healthcare system. These factors created a solid foothold for the intrapreneurial venture within the organization, allowing the project team to pursue ongoing innovation and improvement activity throughout the implementation phase. By physically establishing an ECHO^®^ hub, the organization created an internal safety net with formalized free space and endowed resources to pilot the innovation. This was guaranteed through the ICIF grant funding and additional ongoing investment streams to pursue the expanded use/testing of the ECHO model™. *‘[ECHO] created a seed for uptake more broadly, because the [ECHO panel experts] know that cohorts [of learning participants] can go and say, “Oh, this was good!” you know, and their friends come and do it’* (Participant 2).

#### Create and exploit opportunities for ongoing change (Strategy 7)

The project team created and exploited opportunities for ongoing change throughout the implementation phase by actively pursuing funding and partnership opportunities for expansion and improvement activities. This manifested in multiple grant applications, rapid expansion following the ADHD pilot network launch, ongoing quality improvement activities to enhance the acceptability of the innovation, and pursuit of Superhub designation. These opportunities required the project team to be assertive and agile, capitalize on first-mover advantages to innovate and incubate their new knowledge, and cumulative experience in refining and improving the operation of CHQHHS’s ECHO^®^ Networks. *‘I think that [specialist] cultural change occurs slowly, and as a panel, I think we've developed together. But I think that there is still much work to be done’* (Participant 1). This resulted in an increased uptake and acceptability of the model within and beyond Queensland as both a product for learners and a service for regional communities.

The organization also remained assertive and motivated to use the piloting of the ECHO model™ for first-mover advantage to build on reputational prestige and exploit ongoing opportunities for using the model at scale to achieve more integrated healthcare. *‘We’ve been much more successful in promoting ECHO as something great to participate in amongst allied health providers and nurses… What we have learned is the [salaried] health staff are keen to participate in ECHO in their paid work time. That [context is] important for our new series going forward to understand that. And it’s important for the hubs that we train now that we’re a Superhub so that we can tell them that that makes things a lot easier’* (Participant 3).

#### Extensive and diverse representation/membership at multiple organizational forums/communities (Strategy 8)

Throughout the pilot, the project implementation team developed an extensive and diverse representation/membership at multiple organizational forums/communities. This enabled the team to leverage opportunities, influence partnerships and decision-making, and advocate for the innovation to be embedded to effect more integrated healthcare. *‘It was a case of talking to people who I knew personally or professionally, getting them enthused about it, get them interested, to figure out who those people were and contacting them multiple times’* (Participant 2). This largely involved the medical lead joining multiple peak decision-making forums across CHQHHS to advocate for the ECHO model™’s potential to influence wider improvements to integrate care and workforce development at a regional level. However, the perpetual issue of sustained engagement with specialists seeing value in the innovation was cited by interview participants as remaining an ongoing challenge. *‘We implemented ECHO well with good fidelity to the model and the early series were fantastic, but for some reason it didn’t shift the perception that specialists have of General Practitioners’* (Participant 3).

As a result, the above strategies have highlighted the conditions in which the project implementation team embedded Project ECHO^®^ as a new logic, to achieve more integrated approaches to healthcare within an established public healthcare organization. Some facets identified during the intrapreneurial pilot highlighted where the project team had to sustain efforts to embed the new logic within the organization.

The organizational context and workforce characteristics of the project team, described in this study, have presented new knowledge of how intrapreneurs implemented an innovation within the Queensland public healthcare sector. The implementation of ECHO model™ aligned with the strategies and tactics identified for intrapreneurs by Heinze and Weber to successfully implement a new solution to address fragmentation of healthcare service delivery, professional isolation and low-value care (Gottlieb & Makower, 2013; Mundy & Hewson, 2019). The dimensions and strategies/tactics have showcased exemplars of intrapreneurism that were specific to the Project ECHO^®^ pilot within CHQHHS.

## Discussion

Intrapreneurship is needed in the public healthcare sector to assist with the specific challenges associated with increasing costs of service delivery, resource scarcity and geographic barriers (Tuerk, 2015). These challenges create a landscape where the public healthcare workforce faces growing professional isolation and vulnerable or at-risk patient populations experience inequities in accessing quality services (Arora et al., 2010). Organizations in the healthcare sector have adapted to virtual platforms to sustain and support the learning needs of their workforce, which has become clearly evident during the COVID-19 pandemic. This result has seen innovative telementoring approaches such as Project ECHO^®^ being implemented to deliver and scale emerging knowledge and best practices through case-based learning. As identified in this research, the main finding was that the Project ECHO^®^ pilot implementation was considered to be an exemplar of an intrapreneurial new business innovation. The pilot demonstrated strong alignment to current understanding in the literature as a new business within the organizational and sectoral context of Queensland, Australia (Altinay, 2004; Antoncic & Antoncic, 2011; Antoncic & Hisrich, 2001, 2003; Drejer et al., 2004; Heinze & Weber, 2015). The subsequent findings were that the implementation of Project ECHO^®^ within CHQHHS aligned with the strategic/tactical approaches recommended by Heinze and Weber (2015) to successfully embed the innovation within a large-scale public healthcare organization as a way to achieve more integrated healthcare within the Queensland context (Heinze & Weber, 2015).

This study found that intrapreneurism in the healthcare context was useful to effect cultural change amongst tertiary paediatric clinicians who participated as ECHO^®^ Network panellists (content experts) in the early adoption phase of the new business. It was also found that frontline professionals across multiple other sectors accepted the ECHO^®^ Networks, as virtual communities of practice, as an innovative new product/service to effect practice change. This research identified and expanded on evidence suggesting that public sector organizations could benefit from exploring more intrapreneurial approaches to change/innovation given the adoption and embedding of innovations like the ECHO model™ within CHQHHS (Falola et al., 2018; Guven, 2020; Neessen et al., 2019; Park et al., 2014). The key strategies highlighted by Heinze and Weber (2015) were overlaid to understand and contextualize tactics employed by the project team throughout the implementation of the new business innovation to ensure it was successfully embedded within the organization.

When considering the concept of intrapreneurism, or entrepreneurism within an existing organization, scholars refer to intrapreneurially minded individuals on a continuum of entrepreneurship, despite their activity occurring as employees within established organizations. The individual participants in intrapreneurial ventures can flex along a continuum ranging from less entrepreneurial, through to more entrepreneurial (Antoncic & Hisrich, 2003). In this study, the project team’s approach when implementing Project ECHO^®^ within CHQHHS aligned consistently as being more entrepreneurial. The research participants consistently identified that the strategies/tactics were employed as emergent opportunities and challenges arose.

As a broader contribution to the workforce of intrapreneurs, more research is warranted to investigate how executive decision-makers can exploit the intrapreneurial workforce as a commodity within organizations to mobilize more disruptive change/innovation (Drejer et al., 2004; Enslin, 2010; Falola et al., 2018; Neessen et al., 2019; Park et al., 2014). Executives should consider and provide tools and opportunities within the organization for intrapreneurs to excel and proliferate (Park et al., 2014). Given the low population rate of intrapreneurs within organizations generally (<5%), tools such as customized business models would enable this workforce to experiment more with innovation and disrupt the status quo in new ways (Bosma et al., 2010; Park et al., 2014).

### ‘Integration Intrapreneurism’—embedding the innovation

This study explored a unique intrapreneurial approach to integrating care by piloting a telementoring model within a public healthcare organization that addressed the evolving needs of the workforce and community at a regional level while organizational expectations to maintain financial sustainability remained pervasive. This was achievable in the absence of conducive financial systems infrastructure to embed the innovation within the organization. The gap in infrastructure was in the form of a suitable business model or financial instrument that could capture and report on the outcomes achieved by using the ECHO model™ to integrate healthcare service delivery at a regional level. In addition to this, the ECHO model™ ethos of “democratizing medical knowledge to serve vulnerable and disadvantaged populations” (ECHO Institute, 2020b) was challenging to quantify or aggregate as a compelling economic narrative to executive decision-makers.

During the pilot, the absence of a business model was offset by to the project implementation team’s proactive and aggressive pursuit of grant and philanthropic funding, and the organization’s executive seeing value in the model’s use at scale across Queensland to build paediatric capacity in the healthcare workforce (Moss et al., 2020). The projected time required to demonstrate the innovation’s value and financial sustainability (> 5 years) was a longer-term sustainability risk to the organization that the executive decision-makers acknowledged and accepted (Hwang et al., 2017; Moss et al., 2020; Oderanti & Li, 2018). Funding instruments used globally across the public healthcare sector remain geared towards activity outputs rather than population outcomes, stemming from an industrial-era production line theory (Clark & Goodwin, 2010) which can limit the perceived value in outcomes-focussed innovations such as Project ECHO^®^. Furthermore, healthcare innovation has generally remained commercially driven towards fast results that align to political cycles and changing financial constraints (Clark & Goodwin, 2010).

This research identified that system funders required a certain level of evidence to adopt new approaches to care that innovated the status quo, particularly when they would likely need to decommission, disinvest in or redesign existing services to accommodate the innovation in the future (Clark & Goodwin, 2010). This study validated the challenge most intrapreneurs face in conveying the innovation’s value proposition to the workforce and system managers (Simmons et al., 2013). The key value proposition that telementoring using the ECHO model™ presented to the healthcare system was that of sharing tacit and implicit paediatric specialist knowledge at scale in a regional context. Across all the CHQHHS ECHO^®^ Networks during the implementation phase, the project team’s commitment to fidelity of the ECHO model™ reduced historical barriers to participation (geography, technology, cost of providing/accessing services) and to learning (mentorship, case-based learning) while seeking to empower frontline healthcare service providers to deliver enhanced services to consumers locally.

During the implementation, the model showed potential for reaching economies of scale in distributing knowledge that resulted in wider access to best practices, reducing rates of low-value care, and increased acceptability in this new way of providing more integrated healthcare across the continuum. However, there was no clear value chain process which could demonstrate how participation in ECHO^®^ Networks provided learners with convenient access to valuable knowledge and mentorship which was directly attributable to enhancing patient outcomes. This was due to there not being specific service/activity item numbers that could be recorded or billed for the learning participant’s time or patient benefits against any Australian healthcare system funding schedules. Further research is necessary to investigate business modelling for Project ECHO^®^ within the healthcare system (Oderanti & Li, 2018).

This study has provided a series of key findings, with the most important being that the Project ECHO^®^ implementation within CHQHHS was an exemplar of integration intrapreneurism in practice. While this was not deliberate at the time, the combination of factors at play across the pilot phase evidenced a consistent alignment of the ECHO model™’s implementation as an intrapreneurial innovation as described by Antoncic and Hisrich (2003). This was further underscored by the project team’s strategic and tactical approaches which aligned to Heinze and Weber’s (2015) study to embed the innovative new logic within the organization.

This study has highlighted that an example of intrapreneurism flourished without any alignment to the regulated financial systems infrastructure in the short term. However, as in the case of the Project ECHO^®^ pilot, intrapreneurial teams would benefit from a tailored business model which could articulate the innovation’s value proposition, longer-term financial sustainability, sources of potential investment/revenue streams, and implementation and evaluation planning support for the organization. There is an increasing value proposition in terms of investment and resource efficiency, as well as patient experience and quality of care improvement where organizations embrace intrapreneurial innovation and disruption. This would enable the expansion of tacit or experiential knowledge creation and virtual dissemination by large-scale organizations (Howkins, 2002; Leadbeater, 2000, 2004; Rifkin, 2000) using innovative models such as Project ECHO^®^. The ECHO model™ showed potential in Queensland to reliably deliver telementoring at a scale that leveraged optimal efficiencies at each point in the healthcare value chain. A tailored business model needs to be designed and would serve as an opportunity for intrapreneurs and system managers to collaborate and invest in ECHO^®^ operations, providing a low-cost, high-impact solution to integrate healthcare service delivery more broadly at a state, national and regional level.

Given the small sample size of interview participants, it is recommended that this study be replicated in other contexts where the ECHO model™ has been implemented. This research would seek to investigate if this study’s findings have been distinct or are representative of other first-moving intrapreneurs seeking to disrupt the status quo (expensive, specialist-level healthcare/other services) as the default standard for routine and/or low-value healthcare service provision, or single-provider interventions (Hwang et al., 2017).

## Conclusion

This study has explored the key learnings of the Project ECHO^®^ pilot implementation in Queensland to provide an understanding of what the project team’s strategies and tactics were during the process to embed a new business innovation, and what factors were consistent with intrapreneurship. These learnings were realized during the implementation and piloting of Project ECHO^®^ as an innovative telementoring model within a public healthcare organization in Queensland, Australia.

Based on a unique dataset of interview transcripts from key project personnel, the authors have been able to map the learnings and milestone experiences of the Project ECHO^®^ implementation in a large-scale, public paediatric healthcare organizational setting as an exemplar of intrapreneurism. The strategic and tactical approaches used by the project implementation team during the pilot phase were analysed against their association with success, opportunities for improvement and failure along a continuum (Antoncic & Hisrich, 2001, 2003; Heinze & Weber, 2015). These learnings can be adapted to other contexts where intrapreneurial project teams are implementing innovations within established organizations across a variety of sectors. The key findings present new evidence suggesting intrapreneurial project teams would benefit from developing or using a tailored business model to ensure their innovation’s sustainability in the longer term to affect the desired change. This research contributes to a better understanding of the strategic and tactical approaches to implementing innovation within a public healthcare organization, with learnings that can be adapted by intrapreneurs in other contexts.

Global events, such as the COVID-19 pandemic, will continue to impact the ways organizations operate and innovate to remain responsive to their regional, workforce and consumer audience needs. Established organizations can position themselves to adapt well to emergent change by fostering environments where intrapreneurs are empowered to innovate and shift existing logic within the system. Change and innovation within healthcare sector organizations could yield regional impact where organizational executives and leaders encourage integration intrapreneurship and the pursuit of opportunities to create value.

## Data Availability

The datasets analysed during the current study are available from the corresponding author on reasonable request.
